# Probabilistic Maritime Trajectory Prediction in Complex Scenarios Using Deep Learning

**DOI:** 10.3390/s22052058

**Published:** 2022-03-07

**Authors:** Kristian Aalling Sørensen, Peder Heiselberg, Henning Heiselberg

**Affiliations:** 1 DTU Security, National Space Institute, Technical University of Denmark, 2800 Kongens Lyngby, Denmark; hh@dtu.dk; 2 Geodesy and Earth Observation, National Space Institute, Technical University of Denmark, 2800 Kongens Lyngby, Denmark; ph@space.dtu.dk

**Keywords:** Automatic Identification System (AIS), deep learning, trajectory prediction, Mixture Density Network (MDN), Long Short Term Memory (LSTM), Maritime Situational Awareness (MSA)

## Abstract

Maritime activity is expected to increase, and therefore also the need for maritime surveillance and safety. Most ships are obligated to identify themselves with a transponder system like the Automatic Identification System (AIS) and ships that do not, intentionally or unintentionally, are referred to as dark ships and must be observed by other means. Knowing the future location of ships can not only help with ship/ship collision avoidance, but also with determining the identity of these dark ships found in, e.g., satellite images. However, predicting the future location of ships is inherently probabilistic and the variety of possible routes is almost limitless. We therefore introduce a Bidirectional Long-Short-Term-Memory Mixture Density Network (BLSTM-MDN) deep learning model capable of characterising the underlying distribution of ship trajectories. It is consequently possible to predict a probabilistic future location as opposed to a deterministic location. AIS data from 3631 different cargo ships are acquired from a region west of Norway spanning 320,000 sqkm. Our implemented BLSTM-MDN model characterizes the conditional probability of the target, conditioned on an input trajectory using an 11-dimensional Gaussian distribution and by inferring a single target from the distribution, we can predict several probable trajectories from the same input trajectory with a test Negative Log Likelihood loss of −9.96 corresponding to a mean distance error of 2.53 km 50 min into the future. We compare our model to both a standard BLSTM and a state-of-the-art multi-headed self-attention BLSTM model and the BLSTM-MDN performs similarly to the two deterministic deep learning models on straight trajectories, but produced better results in complex scenarios.

## 1. Introduction

Maritime activities are affecting all our daily lives with approximately 90% of all cargo being transported by ships, a number that is only anticipated to increase. It is therefore essential to develop surveillance methods applicable for the maritime environment to ensure governmental sovereignty, maritime safety and environmental protection [1]. With the opening of the Northeast Passage to maritime activities such as cargo transport, tourism, fishing, mining and commercial interest for oil, the Arctic ship traffic is expected to increase by 24% by 2027 [2]. Maritime Situational Awareness (MSA) is thus globally becoming increasingly more important, and especially so in the Arctic region [2,3].

For most ships, the Automatic Identification System (AIS) is a compulsory, cooperative maritime communication system used mainly for collision avoidance [4,5,6]. Ships that do not transmit self-reporting data like AIS are called dark ships and these must be observed by other means such as drones, planes or satellites carrying, e.g., imaging sensors [7,8] or Radio Frequency receivers [9]. High-resolution commercial satellite imagery must be scheduled in advance, using both an acquisition time and location and can be acquired down to 3 h after the scheduling. It is therefore essential to have models capable of predicting vessel trajectories 3 h into the future. Considering the uncertainty of trajectories, several probable future locations should be scheduled to increase the possibility of dark ship detection. Similarly, when a dark ship has been located in, e.g., a SAR image, estimating the ship ID increases maritime sovereignty. Using non-probabilistic methods will only allow ships following the most-used routes to be IDed. Conversely, using a probabilistic model allows for both the scheduling of several probable locations and the possible identification of the dark ship(s).

Previously closed-off regions have sparse data suitable for deep learning models and it is therefore excepted that transfer learning must be applied. A trajectory prediction model trained on one region should share many of the same weights as a model needed for another region, i.e., ships behave in much the same manner and have the same general characteristics (sailing straight, not making 8 u-turns in a row etc.), thus motivating the need for training models on other regions.

In this article, we introduce a novel approach to predict maritime traffic using AIS data and deep learning. We implement a Bidirectional Long-Short Term Memory (BLSTM) framework to capture the spatio-temporal dependency of the historical ship routes, similar to current state-of-the-art (SOTA) methods. Future ship trajectories are inherently probabilistic as opposed to deterministic and the novelty lies in our method of predicting the trajectories where, instead of predicting the deterministic location, we model the underlying distribution as a multi-dimensional Gaussian using a Mixture Density Network (MDN) architecture whereafter we sample from the found distribution. We build upon SOTA sequential trajectory prediction methods by framing the problem using Bayesian probability. This allows us to predict several probable trajectories to the same input trajectory at an arbitrary number of time steps into the future. Our BLSTM-MDN model can predict several probable outcomes at, e.g., 5 min or 3 h into the future, showing promising results in both simple and complex scenarios.

In Section 2, we present earlier work done on maritime trajectory prediction and thereby introducing SOTA. This leads to Section 3 in which we introduce both the theory and methodology for our improved trajectory prediction model where we combine BLSTM and MDN to perform an iterative multi step prediction. Then, in Section 4, we show the results of our model both quantitatively and qualitatively and compare it with results from a SOTA model, leading to the Conclusion in Section 5.

## 2. Related Work

Vehicle trajectory prediction has been studied greatly, and [10] analyses the different SOTA methods for predicting the trajectories for cars. The same methods apply for ship trajectory prediction, but whereas cars are constrained by both geometry and driving rules which help in reducing the complexity of the problem, ships are not. Ships are only constrained by geometry at few specific locations. Furthermore, environmental conditions such as weather, currents and more influence the future trajectory, greatly increasing the complexity of the problem. Ref. [10] characterizes exiting methods by input representation, output type and prediction method. Ref. [10] further divide each group by different sub-classes in which, e.g., the output type is characterised by a Manoeuvre Intention, Unimodal Trajectory, Multimodal trajectory or an Occupancy Map. Most often, trajectories are modelled as a unimodal output in which only a single deterministic trajectory is estimated. The multimodal mode is then further divided into a static and a dynamic mode, while the dynamic multimodal mode is more representative of real-world problems, it generally suffers from poor convergence and difficulties in exploring different outcomes. To summarise, while a dynamic multimodal trajectory output is more representative, it is rarely predicted for vehicle prediction due to its complexity, and it has been researched even less for maritime trajectory prediction. Ref. [10] describes the SOTA prediction methods, all being Deep Learning methods. They briefly mention non-deep learning models, such as physics-based models or classical statistical models, but conclude that they have poorer results. They divide the methods into Convolutional Neural Network (CNN) based, Recurrent Neural Network (RNN) based and other deep learning methods like Graph Neural Networks (GNN), Fully-Connected Neural Networks and combination of them all. They conclude that LSTMs show superior results for the temporal correlation. For static scenes, CNNs or GNNs shows superior results. In scenarios where image-like data cannot be used, like regional scale maritime-traffic, both CNN and GNN show worse results. Subsequently, they reach the conclusion that SOTA models must use stacked LSTM layers and that for static scenes CNNs must be used. They also conclude that models with multimodal outputs have worse RMSE scores that those with unimodal outputs. For maritime traffic prediction, SOTA models estimate unimodal outputs using stacked BLSTM layers. Only in rare occurrences, with limited regions, have CNN been used. We aim to develop a model capable for predicting multimodal outcomes using stacked BLSTM layers with added MDN. Considering the large region of interest, we do not opt for a CNN based model.

It is only in recent years that much effort has been put into predicting maritime traffic. Especially so since the advent of satellite based AIS receivers and the resulting near-global and frequent AIS coverage. The work on maritime trajectory prediction consist mainly of data-driven models including both supervised and unsupervised machine learning models.

In [11] the regression problem was turned into a classification problem using several supervised machine learning models such as Decision Trees, Nearest Neighbours and Naive Bayes as well as, e.g., a linear support-vector machine. The future region for a ship was predicted instead of the actual location, with only the KNN model showing promising results. In [12,13] they developed the unsupervised Traffic Route Extraction and Anomaly Detection (THREAD) model which has been further developed in, e.g., [14,15]. The THREAD model is able to both detect anomalies and predict trajectories by extracting the trajectory patterns and turning them into way points. In THREAD, the way points are clustered using the DBSCAN method [16]. Predictions are then made by grouping a new trajectory into a class with similar way points, and estimating the future trajectory using the same-class trajectories.

Lately, deep learning models have outperformed classical machine learning models, as was described in [10]. Refs. [17,18] predicted the future location as a class relative to the last location resulting in a probabilistic relative prediction. They argued that a global classification schema was near computationally impossible considering the grid-size needed. Using a relative class, they circumvented this problem, albeit it showed questionable results for operational usage. Researchers have since modelled trajectories by regression models, where they try to predict a single unimodal deterministic location not capturing the inherent probabilistic nature of ship trajectories. These models predict only the most probable location. In scenarios with two routes where one is slightly more probable than the other, the deterministic models will always predict the most probable route [10].

Refs. [19,20,21] all implemented a BLSTM neural network, a deep learning framework able to retain memory, in which they exploited somewhat large amount of historical data to predict the future trajectories by learning how the vessels sail dependant on past trajectories. Each showed promising quantitatively results for only one time step predictions and [19] showed BLSTM models outperforms other recurrent networks like, e.g., Recurrent Neural Networks [22] or unidirectional LSTM [23]. Ref. [21] only used a small data set but reached the same conclusion. Each article neglects to show how the models performs when predicting further into the future than one time step; Most deep learning models predict the trajectory one step into the future, and quantifiable metrics like, e.g., the root-mean-squared-error only shows how well the model performs one step into the future. Ref. [24] implemented a MP-LSTM to perform an iterative Multi-step Prediction(MP). Here, they designed the model to predict the location one time step, $t_{1}$ into the future. The predicted location at time $t_{1}$ is used to make a prediction at time $t_{2}$. It is then possible to predict the location at an arbitrate time, defined by the span of a time step. Ref. [24] showed the advantages of an iterative MP approach as opposed to a direct MP approach. In a direct MP approach, a single prediction is made at an arbitrary time as defined in the model architecture. It is hence not possible to make predictions at, e.g., both time $t_{1}$, $t_{3}$ and $t_{11}$ in a direct MP model. The disadvantage of an iterative MP approach is therefore mainly that it is more difficult to predict the locations far into the future.

Lately, Attention networks [25] has gained much interest in the deep learning community and [26,27,28] have implemented Attention in the schema of trajectory prediction. Refs. [26,27] implemented Attention, with LSTM, for the mutual interaction of self-driving cars and pedestrians, respectively. Ref. [28] used it for regional maritime trajectory prediction. They decomposed the trajectories in a local region into clusters using both a variational encoder/decoder structure and the DBSCAN method. They then learned the features of each cluster using a BLSTM with added Self-attention and argued that the Attention improved predictive capabilities.

Before the advent of deep learning, many experimented with modelling the underlying probabilistic distribution of the trajectories instead of the deterministic location, such as in, e.g., [12] or [29]. In [30,31] they combined the memory capabilities of a LSTM network with the probabilistic-modelling capabilities of a MDN to predict the conditional probability of the future location, and thereby getting a multimodal output as was described in [10]. In [30], they used the method for projectile prediction and in [31] for basketball trajectory prediction.

Not much effort has been put in combining the probabilistic approach in [29] and deep learning methods in [21] for maritime traffic prediction. We therefore attempt to improve SOTA maritime prediction methods as seen in, e.g., [28] by utilising the MDN architecture enabling us to perform a probabilistic iterative multi step prediction, e.g., 3 h into the future.

## 3. Methodology

The prediction of maritime traffic on AIS data is here modelled using a BLSTM network for the spatio-temporal dependency and a MDN to capture the underlying trajectory distribution. Firstly, a brief introduction to deep learning is given. Thereafter, the AIS data processing, in the context of trajectory prediction, is explained.

### 3.1. Deep Learning

In deep learning, a mapping is made between a known input and output. This mapping is made up by intertwined mathematical functions, called neurons, placed in hidden layers between the input and output. These neurons learn to map the output based on the input, by updating their weights through a forward and a backward pass [32]. In the forward pass, the known output is estimated as

(1) $$\hat{y}=f\left(xW+b\right),$$

where x is the input and W the weight matrix of the neurons, describing the relationship between the input and output and the neurons, and b is the bias. The function, f(·) is called the activation function, describing the mathematical relationship between the input and output. By superimposing many activation functions, $\hat{y}$ can be estimated, where it can be shown that all functions can be described using enough non-linear activation functions. In practice, all differentiable functions can be used, albeit few specific types are often used in the literature [32].

The error between the true output y and the estimated output $\hat{y}$, is quantified by a loss function, $L(y,\hat{y})$. An often used loss function is the root-mean-squared-error function (RMSE)

(2) $$L\left(y,\hat{y}\right)=\sqrt{\frac{1}{l}\sum_{i=1}^{l}{\left(y_{i}-{\hat{y}}_{i}\right)}^{2}},$$

where *l* is the number of observation over which the forward pass is made, formerly called the batch size. Better estimations, $\hat{y}$, are found by minimising $L(y,\hat{y})$ wrt. to both W and b in the backward pass using an *optimiser* as

(3) $$\begin{array}{cc} W_{update} & =W_{old}-\eta \frac{\partial L(y,\hat{y})}{\partial W_{old}},\\ b_{update} & =b_{old}-\eta \frac{\partial L(y,\hat{y})}{\partial b_{old}}. \end{array}$$

By updating W and b with a step size called the learning rate, η, the deep learning network learns the mapping from inputs to outputs. With more layers, increasingly more complex mappings can be made, such as in a Fully Connected layer (FC) in which the output from each neuron in one layer is the input to all the neurons in another layer [32].

### 3.2. Long Short Term Memory Networks

In temporal problems, it is a necessity to map the output from not only the current input, but the output at previous states, i.e., to retain memory. A LSTM network is a neural network with connections pointing backwards, meaning that the output from a neuron is sent back to the neuron itself in the next forward pass [32]. This is effectively a memory state for the neuron, enabling it to learn based on past events. The manner of which the LSTM learns, and weights are updated, is controlled by two states; the hidden state at time *t*, $h_{t}$ and the cell state at time *t*, $c_{t}$, both shown in Figure A6 in Appendix C. Whereas the hidden state is used to make a prediction and can be seen by all other hidden states, $c_{t}$ is internal and can only be seen by the specific neuron and is consisting of four internal cell *gates*; the *output* gate, $o_{t}$. The *input* gate, $i_{t}$. The *forget* gate $f_{t}$ and the *gate* gate, $g_{t}$

(4) $$\begin{array}{cc} i_{t} & =sigm(W_{i}h_{t-1}+U_{i}x_{t}+b_{i}),\\ f_{t} & =sigm(W_{f}h_{t-1}+U_{f}x_{t}+b_{f}),\\ o_{t} & =sigm(W_{o}h_{t-1}+U_{o}x_{t}+b_{o}),\\ g_{t} & =\tanh\left(W_{g}h_{t-1}+U_{g}x_{t}+b_{g}\right). \end{array}$$

With sigm(·) being the Sigmoid function and tanh(·) the Hyperbolic tangent function, each acting as an activation function described in (1). Here, $W_{i}$, $W_{f}$, $W_{o}$ and $W_{g}$ are the weights flowing between the gates from the hidden states and $U_{i}$, $U_{o}$, $U_{f}$ and $U_{g}$ the weights for the input and $b_{i}$, $b_{f}$, $b_{o}$ and $b_{g}$ the biases. The sigm(·) function will scale the input value to lie between 0 and 1, and if sigm(x)=0, nothing is passed on. If sigm(x)=1, everything is passed on. The tanh(·) function will scale the input values to lie between −1 and 1, allowing neurons to have negative values. In the forward pass, the cell state, $c_{t}$ is computed using the gates at each time step and the hidden state, $h_{t}$ using the cell state as

(5) $$\begin{array}{cc} c_{t} & =f_{t}\cdot c_{t-1}+i_{t}\cdot g_{t},\\ {\hat{y}}_{t} & =h_{t}=o_{t}\cdot \tanh\left(c_{t}\right). \end{array}$$

By combining (4) and (5), it is possible for a LSTM to better avoid the vanishing and exploding gradients [33], often seen in Neural Networks by better modelling the information that flows through the network - it can learn if and how much to remember using the Sigmoid and Hyperbolic tangent activation functions and their described characteristics. In the backward pass, the weights and biases are updated similar to (3), in a method called Back-Propagation Through Time. Here, the loss is a superposition of the losses calculated on the gradients flowing backwards; The gradients are calculated on the last observation in a sequence. These gradients are then used to calculate the gradients of the second to last observation in a sequence etc. For the first time step in, e.g., (5), an initialisation must be made of $h_{t}$. Often, this is chosen as zeroes. In a BLSTM [32], two symmetrical LSTMs are placed in opposite directions with the first LSTM going in the forward direction and the second LSTM going in the backwards direction. BLSTM has been shown to outperform unidirectional LSTM, [19]. For an illustrations of a LSTM cell, the reader is referred to Appendix C.

### 3.3. Mixture Density Network

For most deep learning models, it is assumed that a target can be found deterministically from the given input, as seen in (1). For problems in which a specific input can generate several probable outputs, for instance in maritime traffic prediction, deterministic methods falls short. In a MDN, the underlying joint probability is modelled [34,35]; Instead of predicting the conditional average, as in (1), the conditional probability of the output, conditioned on the input is found by

(6) p(x,y)=p(y|x)p(x).

Since p(x) is independent of the target, only the conditional probability is modelled. This is done by approximating the probability as a mixture of several known probability density functions (PDF) as

(7) $$p\left(y|x\right)=\sum_{m=1}^{M}\alpha_{m}\left(x\right)\phi_{m}\left(y|x\right),$$

where *M* is the number of mixtures, $\alpha_{m}\left(x\right)$ is the mixture weight for the *m*’th mixture normalised using the softmax function, and $\phi_{m}\left(y|x\right)$ the individual mixture component, modelled as a known PDF, depending on the problem definition. Each component is modelled as a Gaussian PDF, i.e.,

(8) $$\phi_{m}\left(y|x\right)=\frac{1}{{\left(2\pi \right)}^{f/2}\sigma_{m}{\left(x\right)}^{f}}\exp\left(-\frac{||y-\mu_{m}\left(x\right){\left|\right|}^{2}}{2\sigma_{m}{\left(x\right)}^{2}}\right),$$

in which *f* is the number of target features and $\mu_{m}\left(x\right)$ the mean vector of the mixture features, representing the location parameter for the *m*’th mixture, and $\sigma_{m}\left(x\right)$ the standard deviation, representing the scale parameter. By designing an appropriate deep learning network, the mixing parameters of the conditional probability can be computed using backpropagation as in (3). Employing a MDN architecture makes it possible to predict $\hat{y}$ by taking either the most probable estimate or by sampling from the *M*’th dimensional distribution and thereby getting a probabilistic target, $\hat{y}$ instead of a deterministic target [34].

### 3.4. Data

Each AIS message contains static ship related information such as an identification number (MMSI), it further includes voyage related information like destination and estimated time of arrival and lastly it includes dynamic information [4]. The dynamic information is passed to the AIS transmitter automatically by an, e.g., an Inertial Measurement Unit aboard the vessel and is sent regularly with a frequency depending on among else, location and vessel status, and carries information such as latitude and longitude, speed over ground (sog) and course over ground (cog). Only the dynamic information from the messages, along with the MMSI number, is used in the following due to the many errors (intentional or unintentional) in the crew-entered static and voyage related AIS data [4,36].

#### 3.4.1. Data Pre-Processing

Each message is acquired chronologically and all messages should therefore firstly be processed into trajectories for each ship. This is done by grouping the trajectories by MMSI number. Each full vessel trajectory is then divided into sub-trajectories, corresponding to when the ship stops for a time period longer than 3 h, e.g., when mooring, where only sub-trajectories with a minimum of 50 messages are used. The dynamic data are thereafter resampled and interpolated using the piece-wise cubic-spline interpolation as in [37], with a resampling time of 5 min. A filtering of the sub-trajectories is then made to remove erroneous data including nonphysical and unlikely values such as NAN values and messages with sog higher than 35 kn.

#### 3.4.2. Data for Training

A sub-trajectory is represented by a dynamic tensor with four features, i.e., f=4

$$\begin{array}{cc} x^{\left(j\right)} & =[{\mathrm{lat}}^{\left(j\right)},{\mathrm{lon}}^{\left(j\right)},{\mathrm{sog}}^{\left(j\right)},{\mathrm{cog}}^{\left(j\right)}], \end{array}$$

where $x^{\left(j\right)}$ is the *j*’th sub-trajectory in which each element is a vector of length $T_{j}$ > Minimum messages and j=1…N, where *N* is the total number of sub-trajectories. Considering the uncertainty in especially Satellite-AIS, **lat** and **lon** are both rounded to nearest 0.001${}^{{}^{\circ}}$. This is also done to make it easier for the model to predict future locations. Similarly, **cog** and **sog** are rounded to 0.1 kn and 0.1${}^{{}^{\circ}}$, respectively.

**Figure 1:**
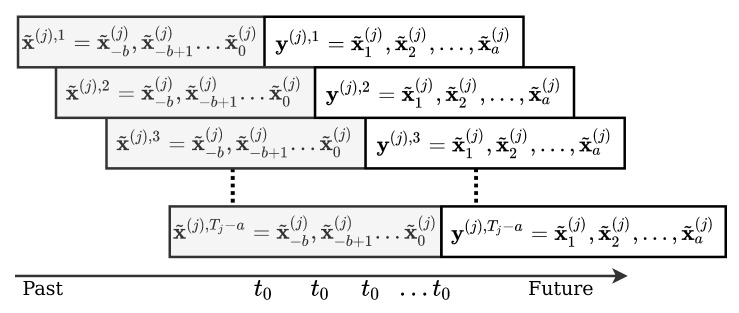
Generating samples and targets from Automatic Identification System (AIS) data using the sliding window approach here shown for a single sub-trajectory, ${\tilde{x}}^{\left(j\right)}$ of length $T_{j}$. Samples, ${\tilde{x}}^{(j),1}\ldots {\tilde{x}}^{\left(j\right),T_{j}-a}$, of size bxf and targets of size axf are made. With the sliding window approach, time $t_{0}$ shifts with the window.

The data are then made suitable for a neural network; Firstly, the data are split into a training and a testing set using a 8:2 split partitioned on the MMSI number, whereafter a final training and validation set is made by making a 8:2 split of the training set. The model is thus trained on the training data, as described above, and validated on the validation set. Each dynamic attribute vector in the entire data set is standardised using the zscore employing the parameters found from only the training set. The zscore standardisation is given by [32]

(9) $${\tilde{x}}^{\left(j\right)}=\frac{x^{\left(j\right)}-\bar{x}\left(x_{train}\right)}{S(x_{train})},$$

with $\bar{x}\left(\right)$ being the sample mean of each feature and **S**() the sample standard deviation. This will give the different features the same importance, even if **cog** has unscaled values larger than **sog**. The samples and targets for the network are then made by adopting the sliding window approach as illustrated in Figure 1. From a sub-trajectory, ${\tilde{x}}^{\left(j\right)}$, of length $T_{j}$, samples are found by taking *b* consecutive time steps. The targets are then the data for the *a* next time steps.

By having a=1, an iterative multi step prediction is made, in which the model predicts only the data for the next time step, which can be used to forecast further. I.e., after *b* predicted time steps, all inputs to the model will be predicted values from the model itself. In this manner, both samples and targets are found using the AIS data. A direct multi step prediction was also experimented with. Not only did this give worse results, it also resulted in less flexibility in predicting locations at several different time steps, as briefly mentioned in Section 2.

### 3.5. Model

**Figure 2:**
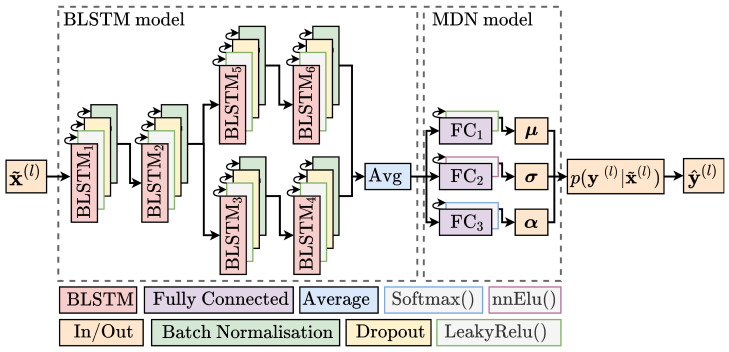
Proposed Deep Learning model with the Bidirectional Long Short Term Memory (BLSTM) model parameterising the temporal dependency and the Mixture Density Network (MDN) model describing the underlying distribution: The input to the BLSTM layers, ${\tilde{x}}^{\left(l\right)}$ are batches of *l* same-size samples shown in Figure 1. Six BLSTM layers are used, each followed by a dropout layer and Batch Normalisation. Three Fully Connected (FC) layers are used to parameterise the features of the Probability Density Function (PDF). The PDF is then used to predict the target for each sample in the batch, ${\hat{y}}^{\left(l\right)}$.

The predictive capabilities are acquired by six BLSTM layers that can capture the spatio-temporal dependencies. The BLSTM layers learns the historic routes, and therefore also how to make predictions based on not only the current trajectory but also past trajectories of similar characteristics. Moreover, dropout layers are used after each BLSTM layer to avoid overfitting [38]. In order to have a probabilistic model, as opposed to a deterministic model, a MDN layer is applied as the final layer. Batch normalisation layers are further used to normalise the input for each layer with Tensorflow default parameters [39]. The model is illustrated in Figure 2, with its hyperparameters shown in Table 1.

#### 3.5.1. MDN

For the MDN, each mixture parameter is parameterized by a FC layer with a specific activation function, with a size depending on the number of mixtures, *M* and features, *f*. The mean is parameterised by the output from the previous layer using the leaky-Relu activation function. For a Gaussian distribution, the standard deviation has the boundary condition of σ≥0 and is therefore parameterized using a non-negative Exponential Linear Unit activation function [41]

(10) $$\sigma {\left(z_{k}\right)}_{m}=1+\left\{\begin{array}{cc} \left(\exp\left(z_{k}\right)-1\right) & \mathrm{if}\;z_{k}<0\\ z_{k} & \mathrm{if}\;z_{k}\geq 0 \end{array}\right..$$

With $z_{k}$ being the output of the previous layer. The distribution must sum to 1, and the mixing parameter is therefore a FC layer with the softmax activation function, i.e., $\sum_{m=1}^{M}\alpha_{m}=1$.

#### 3.5.2. Optimiser

To reduce the risk of local minima in the model, translating to some trajectories being well predicted, and others less so, a cosine annealing is added onto the Adam optimiser with Tensorflow default values [39,42]. An initial learning rate, $\eta_{init}$ is chosen, whereafter η follows the cosine function with an allowed range of $\left[\eta_{min},\eta_{max}\right]$ and period of $E_{i}$. In this manner, training is continuously restarted with increasingly better weights in a method called warm-restarts [42].

#### 3.5.3. Loss Function

**Figure A1:**
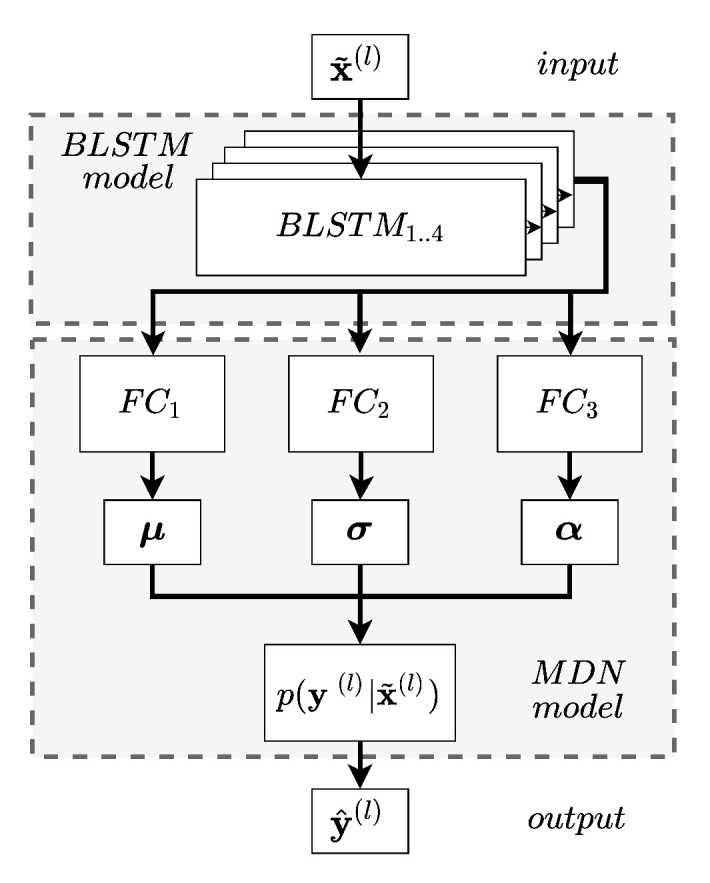
Deep learning model used to estimate the number of mixtures.

The model should construct the mixtures such that the conditional probability of the target y, conditioned on the input x is maximised. The objective is *not* to minimise, e.g., the RMSE as this would *only* minimise the conditional average, neglecting information gained from the standard deviation. Assuming the samples in each batch are independent, the likelihood can be described as

(11) $$L=\prod_{q=1}^{l}p\left(y^{q},x^{q}\right)=\prod_{q=1}^{l}p\left(y^{q}|x^{q}\right)p\left(x^{q}\right),$$

with $x^{q}$ being the *q*’th sample and *l* the total number of samples in the batch. By maximising (11), better values for the mixture components in (8) are found. This corresponds to minimising the negative log likelihood (NLL), −ln(L), in which p(x) is neglected [34]

(12) $$L_{NLL}=\sum_{q=1}^{l}\left(-\ln\left[\sum_{m=1}^{M}\alpha_{m}\left(x^{q}\right)\phi_{m}\left(y^{q}|x^{q}\right)\right]\right),$$

where we use M=11; Few mixtures did not allow for enough flexibility in the prediction of several probable trajectories, and too many mixtures were too difficult to train, albeit more mixtures gives lower NLL. In Table A1, the model shown in Figure A1 has been tested with mixture M=1…12, showing decreasing NLL with increasing *M*.

When using a Gaussian distribution, minimising the conditional mean $||y-\mu_{i}\left(x\right){\left|\right|}^{2}$ corresponds to minimising the RMSE. A deep learning model with the RMSE as a loss function hence discard the knowledge from the standard deviation. Therefore, using a MDN and NLL it is possible to predict a vessel to sail both east and west of a landmass.

## 4. Experimental Results and Discussion

AIS data have been acquired using a PostgreSQL database using a Python wrapper for Python 3.8 [43]. For the deep learning models, the Keras front-end library to Google’s Tensorflow back-end library has been used [39,44]. All models have been trained on a Nvidia Tesla V100 SXM2 32 GB GPU, with an automatic termination when the validation loss has not decreased for 50 consecutive epochs, whereafter the model corresponding to the lowest validation loss is used. In Section 4.1, the data set is explained, and in Section 4.2, the results will be shown. Code is available at https://github.com/aalling93/probabilistic-maritime-trajectory-prediction-in-complex-scenarios-using-deep-learning (accessed on 2 February 2022).

### 4.1. Data Set and Model Setup

**Figure 3:**
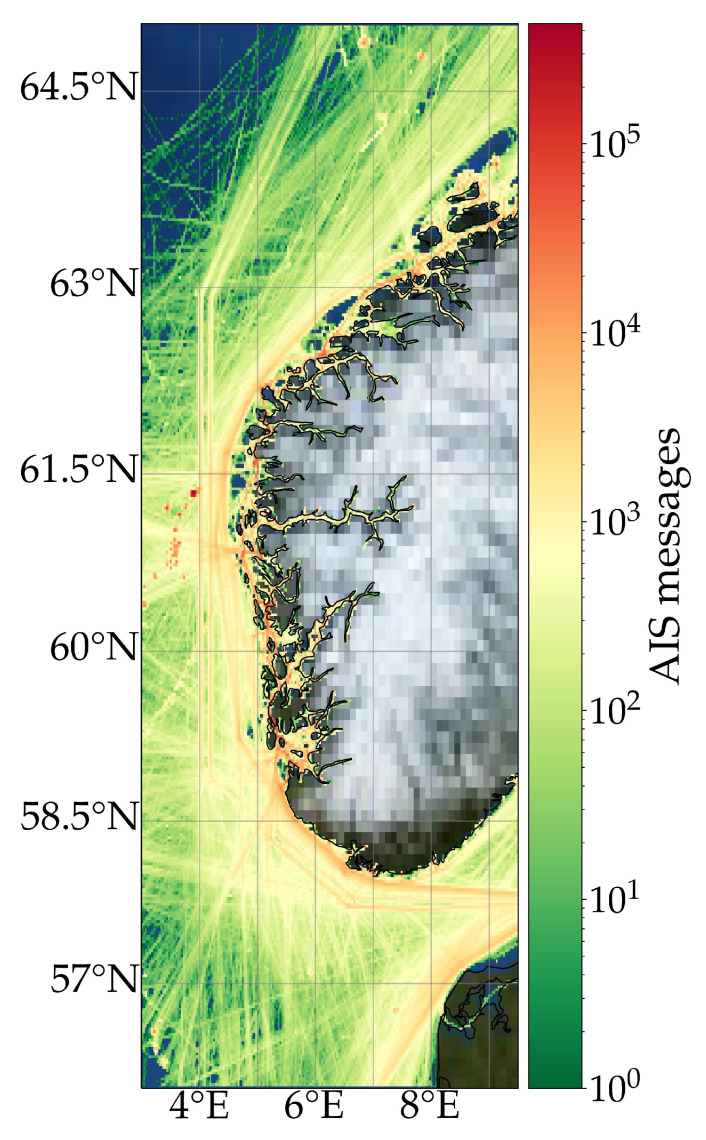
Heatmap of pre-processed cargo ship AIS data near Norway with high density corresponding to red colours. The red lines corresponds to the most used sea lanes. The red dots northeast of Norway corresponds to, e.g., oil platforms.

Raw AIS data transmitted by cargo ships, originally captured from exactEarth satellites, have been retrieved from Gatehouse Maritime’s servers from the region shown in Figure 3, with an area ≈320,000 sqkm. Here, the colours correspond to the normalcy of the cargo trajectories, with warm colours corresponding to often used routes. Data from the summer months of June to August were acquired from 2016, 2017, 2018 and 2019.

For the entire time span, 113,788,768 AIS messages from 10,238 distinctive cargo ships are acquired. Cargo ships are more difficult to model as opposed to passenger ships, but less so than fishing vessels, and are consequently used to prove the model’s effectiveness. The parameters for the data pre-processing can be seen in Table 2. Following the AIS data pre-processing, 3631 different vessels remain. The data have been split such that the MMSI in each data sets are unique for that set, i.e., the model has never seen the ships in the testing set.

### 4.2. Model Evaluation

The model is evaluated both quantitatively and qualitatively.

#### 4.2.1. Quantitative Evaluation

**Figure 4:**
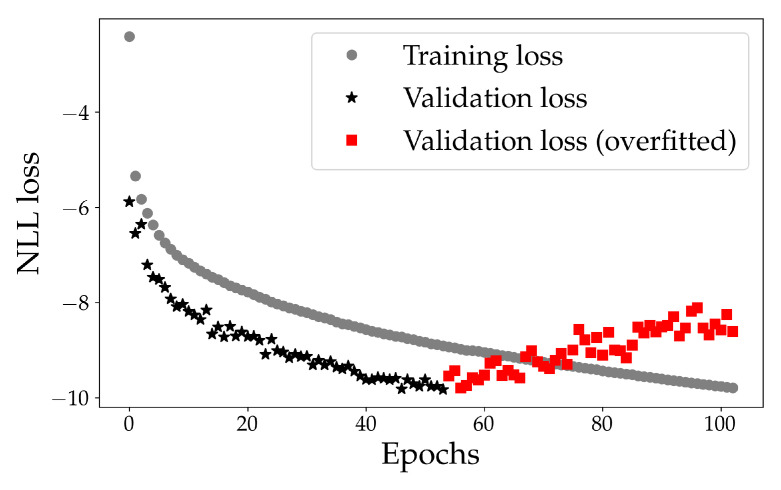
Training and validation negative log likelihood (NLL) loss for the model shown in grey circles and black stars, respectively. The red squares (from validation set) corresponds to when the model starts to overfit the data. The final model is taken from epoch 54.

In Figure 4, the training- and validation NLL loss for the model is shown. It can be seen that the model learns an underlying distribution of the data with a decreasing NLL. Note that there is no lower bound of the NLL.

At epoch 54, the validation NLL loss increases. This is due to overfitting which often occurs when training deep learning models: For a MDN model, this could correspond to specific routes being over-estimated, such as the high-density routes as seen in the normalcy map in Figure 3. The validation loss is lower than the training loss. This is due to the rather high regularisation (dropout) applied during training, but not so during inference, as described in the official Keras documentation [45]. This can be related to the positive impact of the turned off neurons.

In Table 3, each NLL loss is shown. The testing loss for the model (terminated at Epoch 54) is comparable to both the validation loss and training loss. The even lower testing loss could be due to a more representative data set, i.e., that the trajectories in the test set are more similar to the training set than what the validation set is. We tried to avoid this dependency between the data sets by ensuring that the MMSI is unique in each set, i.e., the MMSI in the testing set is not the training set.

**Figure A2:**
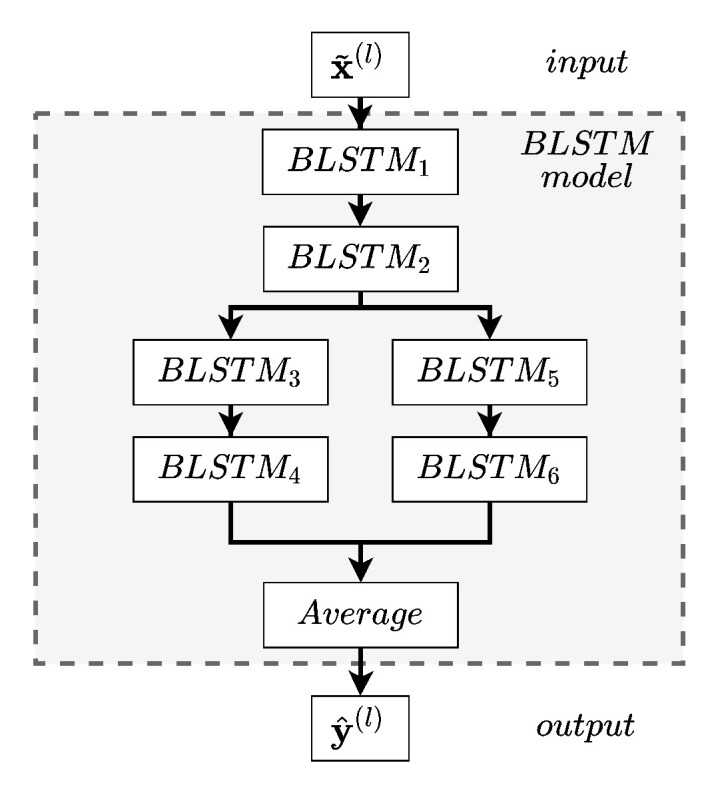
BLSTM model: Same model as the proposed MDN model, without the added MDN layer. RMSE loss function has been used.

**Figure A3:**
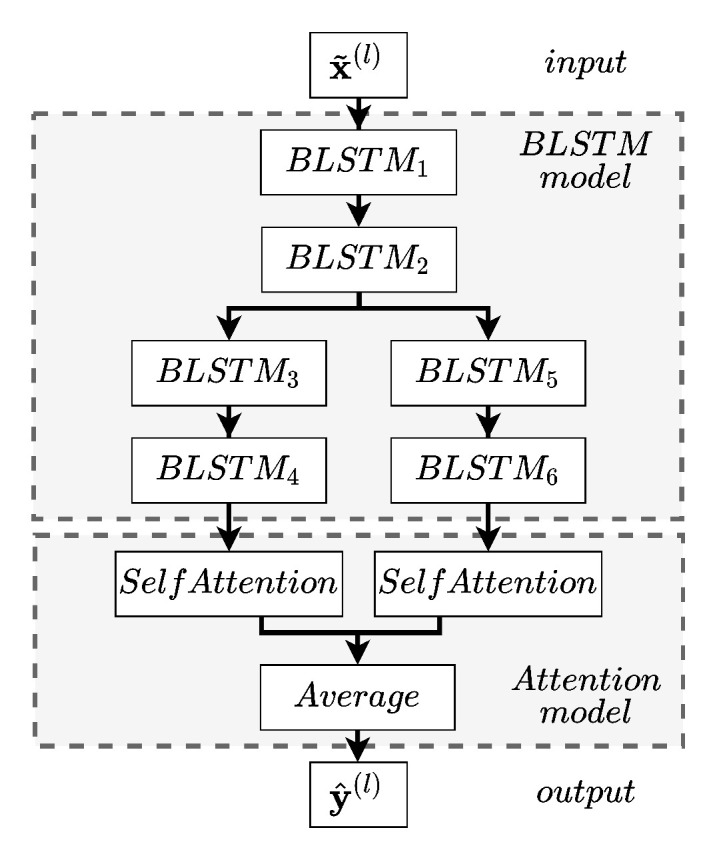
BLSTM with Multi-headed Self-attention model. RMSE loss function has been used.

**Figure A6:**
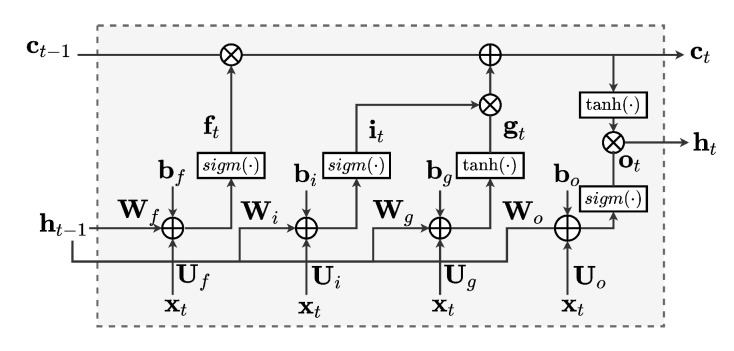
Illustration of the Long Short Term Memory cell. Here, $f_{t}$ is the forget gate, $i_{t}$ the input gate, $g_{t}$ the gate gate and $o_{t}$ the output gate, each with input weights U, hidden state weights, W and biases, b. ⊗ it the multiplication operator and ⊕ the addition operator.

In Table 4, the results from the implemented BLSTM-MDN model is compared with the results from a standard BLSTM model shown in Figure A2 and a SOTA BLSTM model with Multi-headed Self-attention shown in Figure A3. Trajectories in the test set is predicted 25, 50 and 75 min into the future, and the corresponding absolute distance error for the trajectories at each time step is calculated in kilometres using the Haversine function. The Multi-headed Self-attention improves the predictive capabilities of the BLSTM model and is similarly slightly better than the BLSTM-MDN model. This is expected since the MDN will predict several probable trajectories, i.e., reducing the NLL instead of the RMSE.

The more probable routes the worse BLSTM-Attention results, whereas the BLSTM-MDN can predict probabilistic for all different routes. With 10 equally likely routes, the BLSTM-Attention model will predict the same route every time, wheres the BLSTM-MDN can predict each with the same probability and thus return all 10 routes.

#### 4.2.2. Qualitative Evaluation

It is important to show how the model performs when doing an iterative multi-step predictions, i.e., using predicted locations for future predictions, and thereby better simulating an operational scenario.

**Figure 5:**
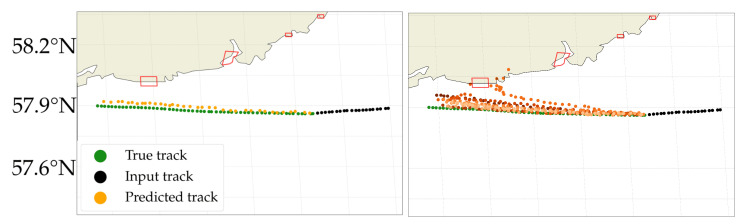
Predictions made with the BLSTM-MDN model. (**left**): A simple cargo vessel trajectory with input samples shown in black, true future trajectory in green and the predicted trajectory in orange. Harbours are illustrated as red polygons with the harbour of Mendal being the leftmost polygon. The model can satisfactorily predict simple trajectories. (**right**): 10 different sampled predictions 36 times steps into the future (3 h), shown in a shade of orange. The predictions mostly follow the true track shown in green, with one future trajectory heading to the harbour of Mandal.

To the left in Figure 5, we have illustrated the capabilities of the model more intuitively using a trajectory from the unseen testing set, with a cargo ship sailing south of Norway in the western direction. Here, the black points are the 20 time steps input equivalent to 100 min of sailing. The orange points are the iterative multi step predictions 36 time steps into future (3 h), sampled one time, with the corresponding true trajectory illustrated in green and nearby harbours as red polygons. At every time step a single location is sampled, resulting in slightly different outputs every time the same input is sampled. After 20 predictions, the predicted trajectory is fully based on historical data; The model has not seen any auxiliary data like shorelines or harbour locations and has therefore learned the trajectory using only past trajectories.

The model has learned how to predict the future trajectory for simple trajectories. The individual predicted location vary slightly from the true location due to the sampling of the distribution. As explained in Section 3, the model has the capability of predicting several probable outcomes, depending on the sampled distribution. If the future, true trajectory (shown in green in Figure 5), was unknown one might expect the ship to sail north towards the harbour of Mandal, Norway.

To the right in Figure 5, 10 different 3 h predictions are illustrated, made from the same input sample. The model primarily expects the cargo ship to continue westward, correctly so. The model also predicted a single trajectory to sail towards the Harbour of Mandal, a harbour known to have cargo ships, whereafter the model fails at future predictions as illustrated with the scattered predictions. This is expected since the model has not seen any training data of cargo ship in harbours.

**Figure 6:**
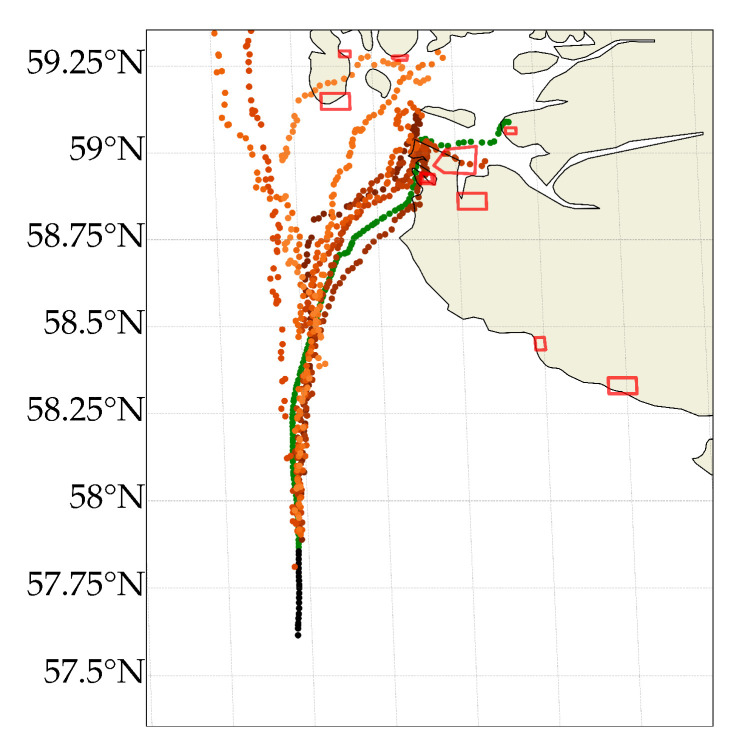
10 predicted trajectories 36 times steps into the future (3 h) in a complex scenario. The model occasionally predicts the wrong trajectory and in rare cases, the future trajectory is predicted to go through, e.g., an island. Two trajectories are predicted to sail northward, the rest are predicted to sail to larger harbours than Tau.

To further visualise the model’s capabilities, Figure 6 illustrates a more complex scenario in which a cargo ship is sailing en route to Tau, Norway—a region with many inlets and harbours. Most of the predicted trajectories avoid the shorelines, with a single trajectory sailing through an island. This illustrates the difficulties when no auxiliary data are provided to the model. We also see that few trajectories are predicted to continue Northward. This is understandable since many cargo ships are sailing south/north in this region. Furthermore, we see that none of the predicted locations are predicted correctly to sail to Tau. Instead, six of the trajectories are predicted to sail to Stavanger, a much larger harbour located close to Tau, explained by many more ships sailing to Stavanger in the training set.

**Figure 7:**
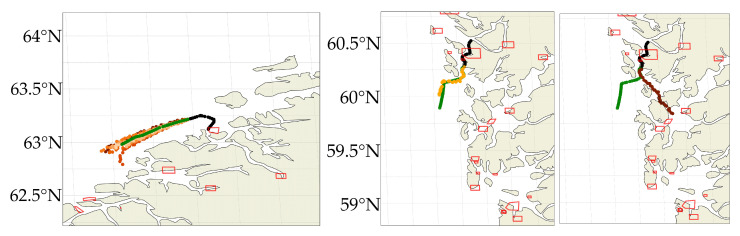
Model predictions with the input sample illustrated in black, the true trajectory in green and the predicted samples in shade of orange. Nearby harbours are shown in red polygons. The predicted trajectory avoid land and follows different probable trajectories.

Similarly, Figure 7 (left) shows ten predicted trajectories from the same input sample. All are predicted correctly in the span of 3 h whereafter most trajectories continue eastward with one sailing south towards the harbours. Figure 7 (mid-right) displays two complex 3 h predictions with same input sample: One follows the true path, albeit at a slower speed, and one sails towards nearby harbours (while beautifully avoiding islands).

### 4.3. Discussion

#### 4.3.1. Model

Our model consists of six BLSTM layers. More advanced architectures were experimented with, such as adding Multi-headed Self-attention layers [25] and encoding/decoding architectures. We did not see a substantial decrease of the NLL loss, compared with the increase of model complexity (when keeping *M* constant). The disadvantage of using the MDN layer is firstly in the choice of mixtures and secondly in the added model complexity; In our model, M=11 was decided upon after experimenting with several discrete choices, see Table A1. With few mixtures, we could not adequately predict several probable trajectories. Conversely, with too many mixtures it is difficult to train. Furthermore, MDN inherently uses FC layers to parametrise the mixing components and this increases the complexity. We modelled the trajectories using a Gaussian distribution, in part because it can be proven that sufficiently many Gaussians can model any distribution [34]. We therefore made the assumption that the trajectories in fact can be modelled using a 11-dimensional Gaussian distribution. Our model showed promising results in a region near Norway in which we had much data. In future work, we will apply transfer learning to the Arctic region.

A prediction at time $t_{1}$ was made by sampling from the modelled distribution. This prediction was then assumed to be correct in the next prediction at time $t_{2}$. If we instead sampled many predictions at time $t_{1}$, this would correspond to Monte Carlo sampling giving the conditional mean similar to the results from a non-MDN model.

In Table 4, we saw the comparison of the different models and how the LSTM model with Multi-headed Self-attention gave the best quantitatively results when analysing the mean absolute distance error. This can be explained using the prediction in Figure 5; the MDN model will occasionally predict different probable routes which increases the mean distance. The trajectory predicted on route to the harbour of Mendal will inherently increase the mean distance error motivating the usage of NLL loss function instead of a RMSE loss function.

**Figure 8:**
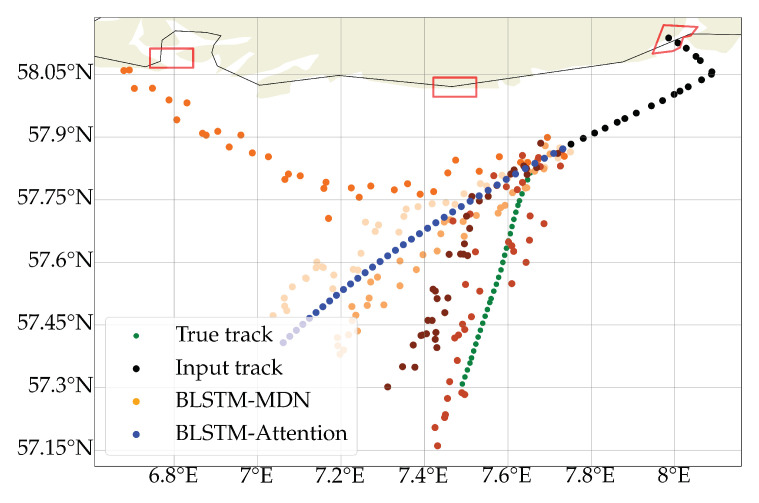
Qualitatively comparison of the predictions from the BLSTM-MDN model (orange shaded points) and the BLSTM-Attention model (blue points). Even if the BLSTM-Attention model had a lower mean distance error that the BLSTM-MDN model, we can see that the BLSTM-MDN model can capture the different possible outcomes better than the BLSTM-Attention model.

In Figure 8, we have illustrated several predictions from a single input trajectory. The blue points corresponds to the 3 h predictions from the BLSTM-Attention model, and the orange shaded points corresponds to 5 different sampled predictions from the BLSTM-MDN model. We can see that the BLSTM-Attention model is predicting the vessel to wrongly sail straight, corresponding to what most ships does. The BLSTM-MDN model has predicted one trajectory to go towards a nearby harbour. It has furthermore predicted two tracks to go in the same direction as the BLSTM-Attention model’s prediction. Lastly, it has predicted two trajectories to head in the true direction and thereby illustrating the power of a BLSTM-MDN model. All routes are probable, but the BLSTM-Attention only predicted the most probable route. It would not have been possible to find the ship in a commercial satellite image if the BLSTM-Attention was used to task a satellite. If a satellite was scheduled using the two most probable areas from the BLSTM-MDN model, the ship would have been found.

#### 4.3.2. Data

For the model, the most important characteristic was firstly the ability to model historic routes (here solved using BLSTM) and secondly to do so using the conditional probability, solved with the MDN. No considerable improvement was seen when trying to improve the network’s hyperparameters. Instead, it was seen that processing the data differently increase the model performance, i.e., trajectory prediction is greatly data driven. It would therefore be worthwhile to analyse the added predictive capabilities when including static/voyage related data; Smaller vessels can sail where larger vessels can not. The Static data can be found using only the MMSI/IMO number and a correct look up table and the voyage-related data can similarly be made using historical data, i.e., determining the destination harbour beforehand.

The data were generally modelled using a regression approach. In the literature, many attempts have been done in predicting future trajectories using a classification schema in which the trajectories are projected onto a grid, see, e.g., [17]. We argue that a regression approach is a necessity for operational solutions. Considering the innumerable amount of future trajectories of vessels, not restricted to roads, a classification schema can not adequately portrait a real world scenario. Furthermore, a classification approach restrict the possibilities of using an iterative multi step prediction approach.

## 5. Conclusions

Maritime trajectory prediction is becoming ever more important with more cargo being transported by sea. Not only is trajectory prediction important for collision avoidance, but also in maritime surveillance and securing sovereignty. Most often, future trajectories are modelled using a deterministic deep learning model in which historic trajectories are used to predict a specific future path. In this article, we introduced a Mixture Density Network (MDN) in combination with a Bidirectional Long Short Term Memory (BLSTM) network and we consequently modelled the underlying distribution of the trajectories and compared the model with a SOTA Attention-based LSTM model. More specifically, we modelled the trajectories using a 11-dimensional Gaussian PDF, enabling us to predict 1 time step into the future with a Negative Log Likelihood loss of −9.97 resulting in a mean distance error of 2.53 km, 50 min into the future. By sampling from this distribution, we showed that we can predict several probable future locations resulting in more realistic predictions. The same past trajectory can result in several probable future trajectories.

## Figures and Tables

**Table 1:** Values of hyperparameters for each part of the model shown in Figure 2 with * corresponding to hidden units in a LSTM, ** is the dropout probability and *** the number of neurons in the respective FC layer.

Hyperparameter	Value	Hyperparameter	Value
BLSTM 1,2,3,5 *	456	$\eta_{init}$	0.0005
BLSTM 4,6 *	256	$\eta_{decay}$	0.7
Mixtures, *M*	11	$\eta_{min}$	$10^{-8}$
FC(1,2,3) ***	(44,44,11)	$\eta_{max}$	0.1
Dropout **	0.3	Batch Size, *l*	3000
Initializer	LeCun N [40]	$E_{i}$	10

**Table 2:** Data pre-processing values. With Look ahead of 1 time step, we are only prediction the location 5 min into the future.

Parameters	Value
Resampling time	5 min
Minimum messages	50
Stop time	3 h
Look back, *b*	20 time steps
Look ahead, *a*	1 time step
Features, *f*	4

**Table 3:** NLL loss for Training, Validation and Testing. The Training NLL is the highest due to a high regularisation during training, but not so during inference. For each sample, a single target with *f* features is given.

Data Set	# MMSI	Targets	NLL Loss
Training	2316	4,810,199	−8.93
Validation	579	1,137,834	−9.55
Testing	726	1,765,351	−9.96
Total	3631	7,713,384	−

**Table 4:** Comparison of the BLSTM Mixture Density Network (BLSTM-MDN) model with a BLSTM model and a BLSTM model with a 2-headed Self-attention mechanism. The distance errors are calculated in kilometers as the mean of all the errors at a specific time step using the Haversine distance function.

Model			
Minutes	BLSTM	BLSTM-Attention	BLSTM-MDN
25	2.2	1.19	1.75
50	3.5	2.42	2.53
75	4.9	4.0	5.07

**Figure A4:**
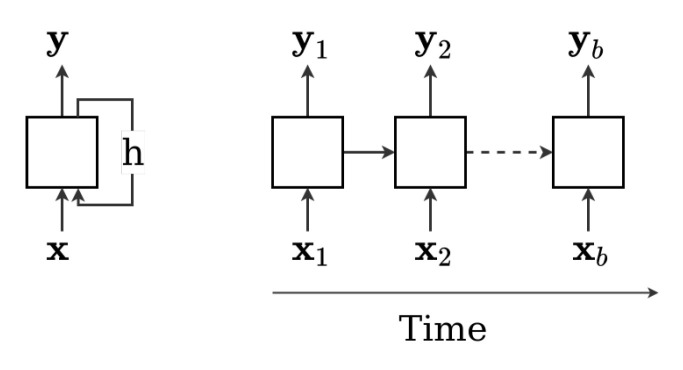
A recurrent neuron (**left**) that is unrolled through time (**right**).

**Figure A5:**
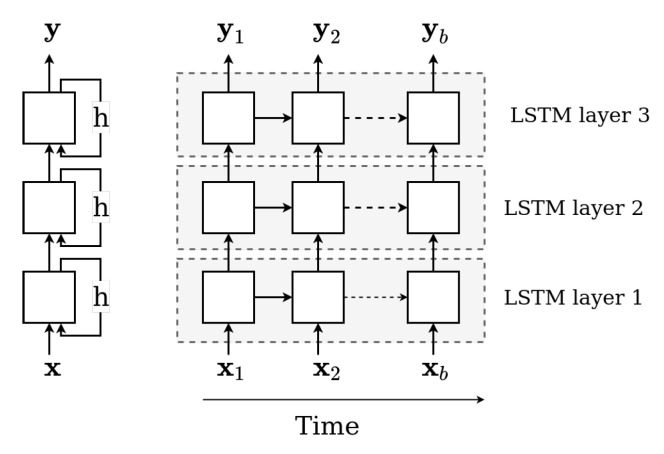
A deep RNN unrolled through time. The output of layer 1 is the input to layer 2. At each time step, each recurrent neuron will receive the input $x_{t}$.

## Data Availability

Not applicable.

## Abbreviations

The following abbreviations are used in this manuscript:

MSA	Maritime Situational Awareness
AIS	Automatic Identification System
BLSTM	Bidirectional Long-Short Term Memory
SOTA	State-Of-The-Art
MDN	Mixture Density Network
THREAD	Traffic Route Extraction and Anomaly Detection
MP	Multi-step Prediction
RMSE	Root-Mean-Squared-Error
FC	Fully Connected
PDF	Probability Density Function
sog	Speed Over Ground
cog	Course Over Ground
NLL	Negative Log Likelihood

## Appendix A. Choice of Mixtures

The number of mixtures, *m* was decided upon after training identical models with m=1−12. The model is illustrated in Figure A1, and is less complex than the proposed model in Figure 2 due to the training time. The models were restricted 120 Epochs, training for approximately 20 h each. The BLSTM-MDN model in Figure 2 took approximately 36 h to train.

In Table A1, the results for each model is shown. The NLL loss is decreasing with increasing Mixtures. Similar to Table 3, the testing loss is smaller than the training loss. Due to the corresponding increase in model complexity, 11 mixtures has been chosen as an executive choice.

**Table A1:** Training, Testing and Validation NLL for Mixture m=1−12.

Mixtures *m*	Training NLL	Testing NLL
1	−5.43	−6.84
2	−5.808	−7.19
3	−6.446	−7.87
4	−6.401	−7.88
5	−6.556	−7.97
6	−6.518	−7.99
7	−6.688	−8.12
8	−6.927	−8.37
9	−6.699	−8.1
10	−7.163	−8.58
11	−6.898	−8.35
12	−7.048	−8.47

## Appendix B. Models for Comparison

LSTM models have long been hailed as the SOTA for trajectory prediction albeit recently, attention models has gained much recognition. The MDN model shown in Figure 2 is thus compared to firstly a standard LSTM model illustrated in Figure A2 and secondly the Attention model illustrated in Figure A3. Both models are comparable to the MDN model. For the Attention model, 2-headed Self-attention layers were used [25]. Excluding the final layers, all BLSTM layers, dropout etc. have the same parameters as the proposed BLSTM-MDN model.

## Appendix C. RNN and LSTM

A Recurrent Neural Network (RNN) is both a specific type of architecture, and a group of similar networks capable of handling sequential data of varying sizes. All RNNs have connections pointing backwards, meaning that the output of a neuron is sent back to the neuron itself in the next forward pass. This is effectually a *memory* state for the neuron, and such a neuron is consequently called a *memory cell* or simply a cell. A simple RNN is illustrated in Figure A4 with a single hidden neuron. Often, many of such neuron are placed in each layer, e.g., 456 are used in each LSTM in this work. At each time step, each recurrent neuron in the layer will receive the input $x^{t}$ at time step *t*, and the output from the neuron at the previous time step $y^{t-1}$.

To the left in Figure A4, we see how the input to the neuron is the activation from the previous layer, x and the neuron’s output from the previous time step, $y^{t-1}$. To the right, we see how this is unrolled through time. The weights for the neuron is shared for all time steps, and the back propagation seen in Equation (3) is therefore for RNNs called *back propagation through time* (BPTT). In the *forward pass*, each sample(a sequence has many samples) is passed through the neural network resulting in a loss as described in Equation (12). This loss is firstly used to calculate the gradients and hence the weight-update for the last time step, like in Equation (3). The gradient from the last time step is then passed backwards. In the second-to-last time step, a gradient will be calculated depending on the gradient and loss from the last time step, and so on. In this manner, the gradients are updated backwards through time, flowing though *each* time step [32]. This makes the RNNs difficult to train as opposed to other networks architectures where a single gradient is found for each sample. For a RNN, it is very difficult to find the best weights for the early time steps due to the BPTT. Furthermore, in deep learning, efficiency is often very important. RNNs are inherently inefficient since the gradients are update through each time step every time. Hence, RNNs are both more difficult to train, and takes longer time to train. Still, due to their performance on sequential data, they are widely used.

A deep, stacked RNN with 3 layers is illustrated in Figure A5. Here we see how the output from one layer is the input to the next, unrolled through time.

A neuron’s values at time *t* is called its *hidden state*, $h_{t}$, given by:

(A1) $$h_{t}=f\left(h_{t-1},x_{t}\right),$$

where $h_{t-1}$ is the hidden state for previous time step. We can therefore see it depends on the input and all the past states(memory). For the first hidden state, $h_{t-1}$ is often initiated as 0. Moreover, if a weight becomes slightly smaller or larger than 1, the BPTT gradients will go towards zero or infinity for long sequences, the *vanishing gradient problem* and *exploding gradient problem*, respectively. The effect of these vanishing and exploding gradients are orders of magnitude worse in RNNs as compared to other neural networks. Due to the BPTT, regular RNNs can in practise only “remember” short sequences in the order of ≈10 samples (the gradients are *multiplied*, and after 10 samples, the gradients of the gradients are either indefinitely small or large, and they can thus not remember further). An alternation has therefore been made to the normal RNN in a neural network called the Long Short Term Memory (LSTM).

The LSTM network is to some extent designed to avoid the problem of vanishing and exploding gradients. This is done by designing how the gradients will flow through the network. The RNN only maintains one state, $h_{t}$. The LSTM maintains two states, the hidden state $h_{t}$ and the cell state, $c_{t}$. The cell state is internal and is only seen by the neuron. This cell is illustrated in Figure A6:

The input to a LSTM are samples of sequences. These sequences can then correspond to any order, e.g., time, sentences etc. The first input for the LSTM is then the first observation, corresponding to $t=t_{0}$, they model will then learn the mapping of going from $t=t_{-1}\to t=t_{0}$ dependant on the initialisation of the model. The model will then learn the mapping of going from $t=t_{0}\to t=t_{1}$ etc. For each time, the weight (both U and W) in the LSTM are shared.

The input to the hidden state $h_{t}$ is the past hidden state $h_{-1}$, as shown in (A1) and the input sample at $x_{t}$. The LSTM hidden cell will then decide how much of the sample to forget, use, and learn from using the gates. This is illustrated as the shaded area in Figure A6. The output of the different gates is passed out of the hidden state, as input to the cell state. Here, the past cell state $c_{t-1}$ is both multiplied and added with the different gates to give a new cell state $c_{t}$. The newly updated cell state, $c_{t}$ is then used to both compute the new hidden state $h_{t}$ and the output of the LSTM (${\hat{y}}_{t}=h_{t}$).
